# Frontier Technologies in Single‐Cell Analysis: Synergistic Fusion of Droplet Printing and High‐Performance Detection System

**DOI:** 10.1002/ansa.70041

**Published:** 2025-09-06

**Authors:** Qi Zhang, Jiahao Li, Yuqing Zhang, Hongsheng Zhang, Cong Wang, Qiang Ma

**Affiliations:** ^1^ Chinese Academy of Quality and Inspection & Testing Beijing China; ^2^ NMPA Key Laboratory for POCT Technology Transforming and Quality Control Beijing China; ^3^ Technology Innovation Center of Analytical Instruments & Equipment Testing and Evaluation State Administration for Market Regulation Beijing China; ^4^ Beijing Key Laboratory of Microstructure and Properties of Solids Faculty of Materials and Manufacturing Beijing University of Technology Beijing China

**Keywords:** droplet, high‐performance detection, printing, single cell

## Abstract

Single‐cell analysis provides critical insights into cellular heterogeneity, dynamic behaviours and microenvironmental interactions, driving advancements in precision medicine and disease mechanism research. However, traditional technologies face limitations due to low throughput, insufficient sensitivity and bottlenecks in multi‐omics integration. Microdroplet printing technology, with its advantages in high‐throughput single‐cell encapsulation, picolitre‐level reaction precision and oil‐free phase contamination avoidance, has propelled single‐cell analysis into a new era of high‐throughput and high‐dimensional resolution through deep integration with multimodal detection platforms. This review systematically elaborates on the theoretical framework, diverse technical systems and multi‐dimensional application scenarios of microdroplet printing technology. It further dissects the deep coupling mechanisms between this technology and multimodal detection platforms such as including mass spectrometry, Raman spectroscopy, fluorescence and ultraviolet detectors, as well as its unique advantages in single‐cell analysis. Such cross‐technology integration has significantly accelerated innovation in fields such as single‐cell drug screening and multi‐omics analysis, marking a significant leap in the evolution of single‐cell analytical methodologies.

## 1 Introduction

In the process of the evolution of life science research from macro organizations to micro individuals, single‐cell analysis technology is reshaping our understanding of life sciences in a revolutionary way [1, 2, 3]. Traditional cell‐based research methods can obtain population whole‐level biological information but inevitably mask the heterogeneity of gene expression, phenotypic characteristics and functional status among cells [4]. With the synergistic breakthroughs in molecular biology, micro‐nano manufacturing and precision detection technologies, the single‐cell resolution research system has gradually broken through the technical bottleneck and has demonstrated unprecedented analytical capabilities in multi‐dimensional analysis of genomics, epigenetics, proteomics and metabolomics [5, 6, 7, 8]. This individualized research not only provides a key breakthrough for revealing the spatiotemporal dynamics of tissue development, the molecular trajectory of disease occurrence [9, 10] and the individual differences in drug response, but also gives birth to a fundamental shift in biomedical research.

As an important breakthrough direction of single‐cell analysis technology system, microdroplet printing technology is reconstructing the experimental topology of single‐cell research by integrating the dual advantages of microfluidic manipulation and precision printing technology [11]. This technology's innovation combines precise liquid control and spatial positioning, utilizing air pumps, electrohydrodynamics or surface acoustic waves to generate and deposit micron‐scale droplets with angstrom‐level precision [12, 13]. Compared with the passive generation mode of droplet microfluidic technology [14], which relies on continuous phase fluid stabilization of droplets, this active droplet generation strategy can greatly improve the efficiency of single‐cell encapsulation by directly manipulating the hydrodynamic boundary conditions at the nozzle while maintaining the uniformity of droplet diameter. Critically, this non‐contact control mode effectively avoids mechanical stress damage to the cell membrane, laying a technical foundation for subsequent high‐fidelity analysis.

In the construction of a single‐cell detection system, microdroplet printing technology forms in situ synergistic integration with multiple analysis techniques through modular structure design, which significantly improves the detection performance. Since there is no need to rely on complex micro‐nano manufacturing processes, the technology has the characteristics of low cost and simplified operation, making it easier to promote and apply on a large scale. Through the precise control of the droplet deposition process and the in situ processing strategy, the interference of the organic phase carrier on the detection signal in the traditional droplet microfluidics can be effectively avoided, so that the signal‐to‐noise ratio of the target molecule detection is improved and the possibility of instrument contamination is avoided. At the same time, the spatial localization characteristics of this technology can achieve accurate matching between the microscale detection region and the excitation unit so as to establish a high‐throughput array analysis platform, which can improve the single‐cell characterization efficiency by several orders of magnitude compared with conventional systems [15].

This review systematically discusses the theoretical framework, technical system and multi‐dimensional application scenarios of microdroplet printing technology and focuses on the deep coupling mechanism between it and multimodal detection platforms (including mass spectrometry [MS], Raman spectroscopy, fluorescence and ultraviolet detectors, etc.), and this cross‐technology integration has significantly accelerated the technological innovation process in the field of single‐cell analysis. Based on these methodological breakthroughs, researchers have been able to achieve high‐precision analysis and multi‐dimensional characterization of single‐cell properties, which has strongly promoted the expansion of cognitive boundaries in the fields of medicine and biology. Such technical synergies not only drive the strategic transformation of the single‐cell analysis paradigm from ‘detectability’ to ‘precise resolution’ but also give rise to cutting‐edge interdisciplinary fields such as spatial omics and dynamic metabolomics and build a new technical reference system for precision medicine and systems biology research.

## 2 Classification of Microdroplet Printing Technology

### 2.1 Piezoelectric and Thermal Drives

**FIGURE 1:**
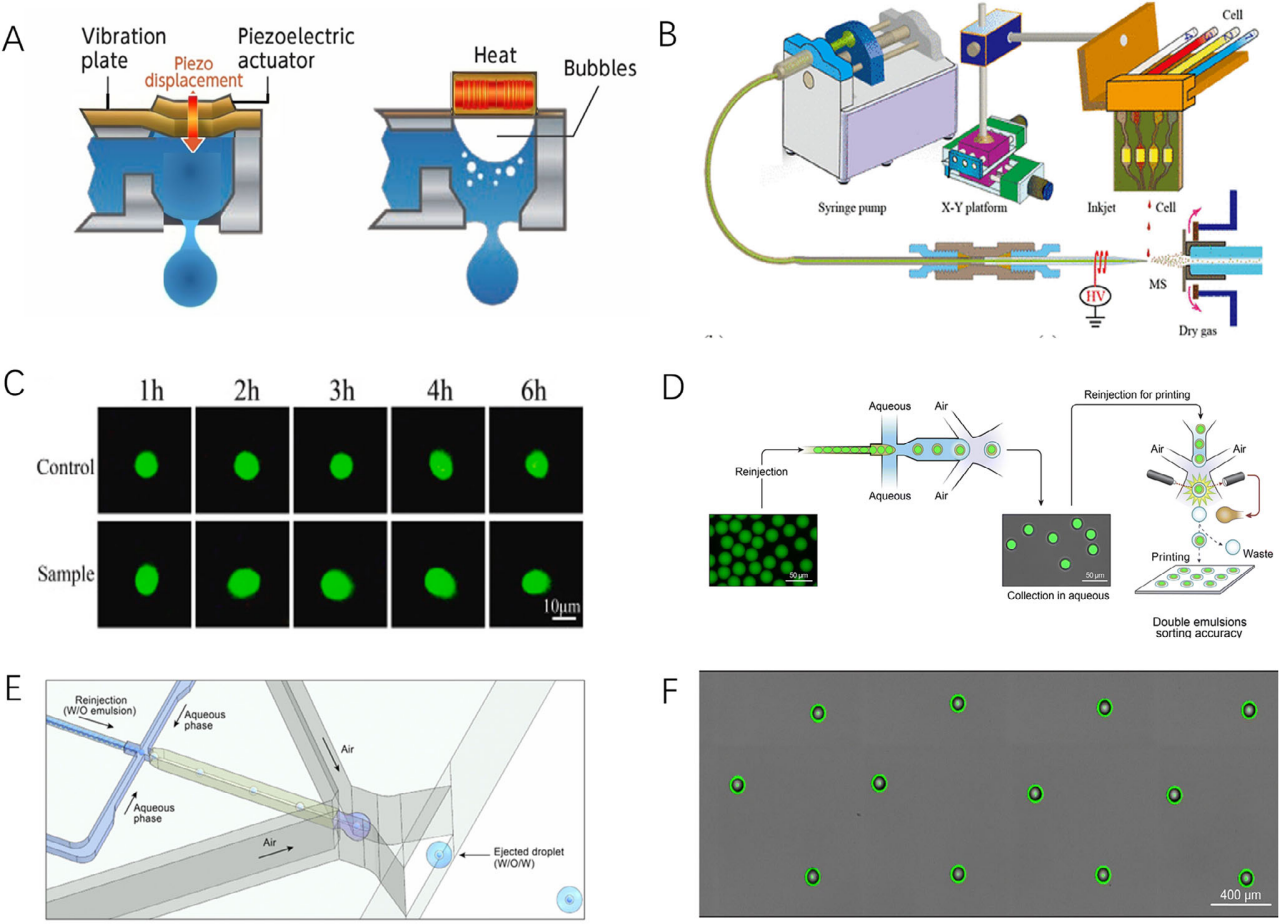
Piezoelectric, thermographic and pressure‐based printing methods. (A) Droplet formation diagram principle in piezoelectric and thermal inkjet systems. (B) Schematic diagram of an inkjet printing single‐cell MS device [18]. (C) Fluorescence map of the dynamic response of carboxyesterase of MCF‐7 cells to 10 mM calcein‐AM in a single cell [19]. (D) Process diagram of printing double emulsion on demand in air [22]. (E) Schematic diagram of a microfluidic device [22]. (F) Printed image of the agent droplet array [22].

Conventional inkjet printing technology is mainly divided into two types: micro‐piezoelectric and hot bubble type: micro‐piezoelectric technology controls the injection of ink droplets through three stages. Before printing, the piezoelectric element shrinks slightly under signal control; the element then extends significantly, pushing the droplets out of the nozzle. As the droplets are about to fly away, the element shrinks again, allowing the ink level to retract precisely to the nozzle. This mechanism ensures that the droplet morphology is complete and the flight path is accurate (Figure 1A). Piezoelectric inkjet printing offers superior droplet control with more uniform morphology and precise positioning, enabling higher‐resolution output. The non‐thermal mechanism prevents ink degradation, broadening material compatibility while extending printhead durability.

Hot bubble inkjet technology (also known as thermal inkjet technology) operates on the principle of high temperature and pressure. The process is as follows: the thin film resistor instantly heats less than 5 µL of ink to more than 300°C in the nozzle area to form a large number of tiny bubbles; the bubbles merge and expand within 10 µs, pushing the ink droplets out; after a few microseconds, the bubble bursts and returns to the resistor and the ink retracts; the surface tension then pulls the new ink replenishment nozzle to complete the cycle [16]. Thermal inkjet printheads offer fast printing speeds and low cost but face challenges: droplet control instability due to bubble‐driven ejection, frequent nozzle clogging from small diameters (requiring strict viscosity control) and cellular shear stress from size‐matched nozzles.

In the realm of biomedical diagnostics and single‐cell research, a plethora of innovative methodologies are emerging, showcasing immense application potential and numerous advantages. Esfandyarpour et al. [17] have ingeniously amalgamated microfluidics, electronics and inkjet printing technologies to develop an ultra‐low‐cost, rapid and miniaturized lab‐on‐a‐chip platform. This platform boasts label‐free, rapid single‐cell capture capabilities, enabling efficient cell manipulation, precise isolation of rare cells and a suite of complex operations, including selective analysis, separation, sorting, concentration, localization, counting and characterization of biological species. Its miniaturized design effectively curtails the consumption of samples and reagents, while the separable architecture of electronic components and the microfluidic chip facilitates the reuse of electronic components, thereby prolonging the device's operational lifespan. Operating based on the inherent physical properties of biomolecules, the platform significantly enhances its clinical applicability by validating cell viability and achieving multiplexing and integration of multi‐step processes with external analyses. Ren et al. [18] have introduced a high‐throughput, on‐demand inkjet printing single‐cell MS method for the rapid screening of biomarkers associated with polycyclic aromatic hydrocarbon (PAH) exposure in KYSE‐150 cells, aiming to elucidate the pathogenesis of PAH‐induced oesophageal cancer (Figure 1B). This method can analyse cells at a throughput of 51 cells per minute, filtering 930 characteristic ions from 3683 detected signals and identifying 91 uniquely significant molecules with potential as clinical diagnostic biomarkers. Furthermore, the study revealed that the behaviour of certain biomarkers in single‐cell and multicellular lipidomics exhibited opposing trends in response to varying PAH concentrations, potentially categorizing KYSE‐150 cells into PAH‐sensitive and ‐insensitive types, offering novel perspectives on PAH toxicity and disease mechanisms. This method's strength in molecular biomarker discovery comes at the cost of complex instrumentation.

However, Lin et al. [19] have developed a convenient single‐cell analysis method by integrating inkjet printing technology with polydimethylsiloxane microchips. This approach achieves a 91% single‐cell encapsulation rate on hydrophobic substrates and precisely controls the number of cells through parameter optimization. Upon connecting the microchip to a glass slide, various parameters of printed MCF‐7 cells, such as enzyme kinetics, cell viability, reactive oxygen species (ROS) levels, apoptosis and proliferation, can be monitored in real‐time (Figure 1C). The high cell viability, proliferative capacity, low ROS levels and low apoptosis rates observed suggest that this method provides a novel avenue for in‐depth single‐cell research. Additionally, analysing the proliferation outcomes of different cell numbers under adenosine triphosphate induction offers fresh insights for diagnosis and pharmacological interventions.

### 2.2 Pressure Drive

Microfluidic droplet printing technology is an advanced printing technology that uses microfluidic technology to control droplet printing, which mainly uses pump pressure as the driving force, utilizing the fine structure and hydrodynamic effects in the microfluidic channel, to control the shape and ejection behaviour of the droplet. The working principle of microfluidic droplet printing technology is as follows: First, the liquid is loaded into the microfluidic channel of the microfluidic chip. Microfluidic channels often have complex geometries, such as microcolumn arrays, microvalves or microchannels, to control the flow of liquids. When the liquid passes through the microfluidic channel, the flow rate and direction of the liquid can be controlled by adjusting the size and geometry of the microfluidic channel, thereby affecting the shape and ejection behaviour of the droplets [20]. Microfluidic technology has emerged as a pivotal research focus in nanoscience, biomedicine, chemistry and mechanical engineering due to its inherent advantages of miniaturization, integration and high efficiency. Representative applications include the fabrication of biomaterials, cellular studies and drug screening.

Fathi et al. [21] used in situ imaging techniques to delve into cell tissue dynamics with picolitre (pL) precision using extrusion‐based microcapillary tips and air deposition. The microcapillary extrusion setup ‘Picodis’ used in this study was demonstrated by generating colouring ink droplets immersed in immiscible media, using 3T3 fibroblasts as a model to clearly demonstrate the deposition process of cell suspension and pre‐aggregated cell pellets. It was further found that the dynamic organization of the cells within the microcapillary tip and the printing and deposition behaviour of the cells as they exited the tip opening were notable, and it was also observed that the movement pattern of the cells dispersed in the medium was unique according to the flow profile and was entirely driven by laminar flow within the narrow tip. This study provides key insights into the limitations of the high‐resolution cell ink extrusion process. Delivering fluids in air is essential for its applications in MS, bioanalysis, and materials synthesis. Although methods for generating liquids in air have been developed, controlled printing of droplets has not yet been achieved. Zhang et al. [22] proposed a method for air‐printing reagents on demand (Figure 1D). In this method, the reagents are pre‐encapsulated in an emulsion and subsequently re‐injected into the device (Figure 1E), generating droplets in a microfluidic nozzle with spatial pattern wetting. The equipment is able to sort the ejected reagent droplets in real time so as to achieve deterministic printing of each droplet and its desired core (Figure 1F). This method provides a versatile platform for the large‐scale construction of a well‐defined array of printed reagent droplets.

### 2.3 Electrohydrodynamic Drive

**FIGURE 2:**
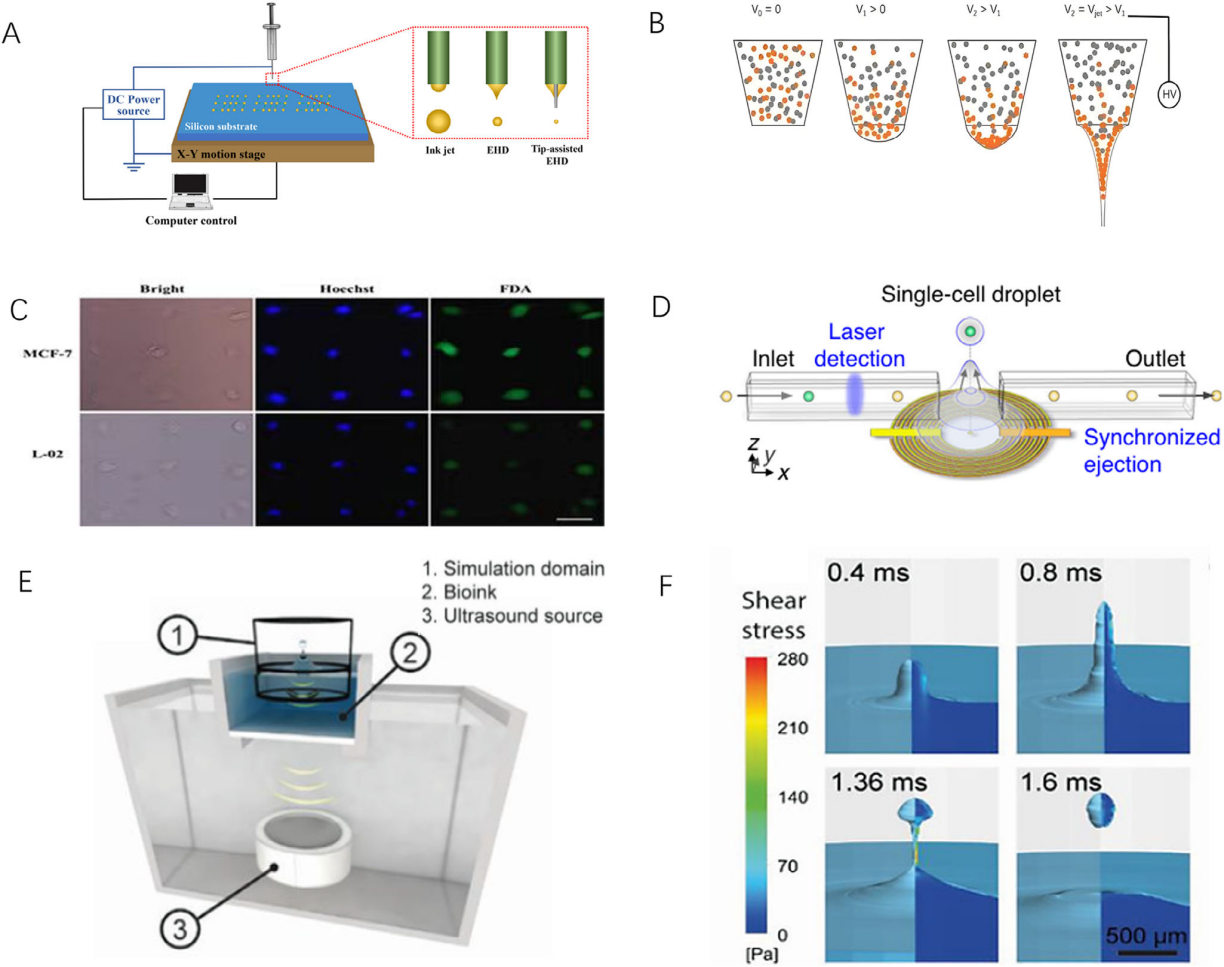
Electric and Acoustic Wave‐Driven Printing Techniques. (A) Experimental setup for electrodynamic printing [24]. (B) Electrodynamic printer diagram. (C) Cell viability test after cell capture [26]. (D) Schematic mechanism of deterministic single‐cell printing by PULSE [29]. (E) Sonic droplet printing Schematic diagram of the device. (F) Double‐exposure image of droplet printing [30].

Electrodynamic printing is an advanced printing technique that uses the coupling effects of electric field and hydrodynamics to control droplet printing [23]. It regulates the movement of ions in the liquid by applying an electric field and an electric current in the nozzle, which changes the shape, velocity and direction of the droplet. The devices of this technology mainly include a voltmeter, an X–Y motorized displacement stage, a syringe pump, a droplet collection substrate and a droplet injection head [24] (Figure 2A). The core principle is to use an externally applied electric field to pull the liquid ink out of the nozzle, rather than being pushed by mechanical pressure, to achieve high‐precision patterning with a resolution greater than 1 µm. When a high voltage is applied between two electrodes, a corona discharge occurs, causing ions of the same polarity as the electrode to move towards the other electrode, forming a space charge and creating a flow of current. The Coulomb force between the ions initiates a dynamic flow of electric fluids, which drives the droplet printing. Specifically, when the printhead of a printing system contains inks with different charges, a voltage is applied between the nozzle and the substrate and the positive (anion) ions gather at the tip of the nozzle of the positive (negative) electrode (relative to the grounded substrate) and form a conical meniscus called a Taylor cone. As the voltage increases, the electrostatic stress gradually overcomes the surface tension and viscosity and finally, the liquid is sprayed from the cone tip onto the substrate, achieving high‐speed, high‐precision and multi‐material printing (Figure 2B).

Electrohydrodynamic jet bioprinting offers ultrahigh resolution (submicron‐scale), minimal cell damage and broad material compatibility, making it ideal for printing delicate tissues (e.g., microvasculature) and sensitive cells. However, it suffers from process complexity, low throughput, stringent bioink requirements, potential cell viability impairment by high‐voltage fields and elevated equipment costs. Wu et al. [25] developed a single‐cell dispenser inspired by electrohydrodynamic jet printing, which demonstrated practical application by sorting and dispensing cells into multi‐well plates for single‐cell polymerase chain reaction analysis. The dispenser combines an optical detection window and a droplet dispensing nozzle with a fused silica capillary for precise droplet generation and single‐cell sorting and dispensing. Through COMSOL simulations and experiments, the system optimizes parameters that affect the droplet partitioning performance, such as the inner and outer diameters of the capillary, the flow rate, the applied voltage and the properties of the solution. Using capillaries with an inner diameter of 100 µm and an outer diameter of 160 µm, small volumes (5–10 nL) of droplets can be obtained, and efficient encapsulation and dispensing of single cells can be achieved. Sun et al. [26] proposed a simple, low‐cost method for constructing high‐resolution patterns on viscous superhydrophobic (SH) substrates with ordinary precision based on inkjet printing as an application case (Figure 2C). This method has significant advantages, such as an SH surface with a high contact angle and relatively high contact angle hysteresis, which can not only obtain high‐resolution dots but also avoid droplet rebound behaviour. The feature size of the printed protein spots is increased to 4 µm, which is much smaller than that of the protein spots used for single‐cell capture; inkjet‐patterned chips with fibronectin inks can capture multiple single‐cell arrays quickly and efficiently with the aid of narrow microchannels. As a proof of concept, the chip has been applied to study real‐time apoptosis in single cells, demonstrating the potential for cell heterogeneity analysis, with a single‐cell occupancy rate of approximately 81% within 30 min on subcell‐sized patterned chips with no significant effect on cell viability.

### 2.4 Acoustic Drive

Acoustic printing is an advanced printing technology that uses the interaction of sound wave propagation and liquid to achieve droplet printing and control. It induces stimulates pressure changes and surface fluctuations in the liquid by creating acoustic vibrations in the nozzle to enable droplet formation and printing [27]. By adjusting parameters such as frequency, amplitude and phase of the sound wave, the size, velocity and printing position of the droplets can be controlled. It functions through the following mechanism: a liquid is loaded into a nozzle, and then sound waves are generated by vibrating a sound transducer. When sound waves travel through a liquid, they cause compression and expansion of the liquid molecules, creating pressure changes and surface fluctuations. These changes cause the liquid to form adjustable size droplets from the nozzle, which are ejected by the radiant pressure of the sound waves. By adjusting the acoustic parameters, the shape, size and velocity of the droplets can be controlled [28]. The acoustic printing technology demonstrates superior performance relative to other microfluidic and positive displacement liquid handling methods through multiple key characteristics: its capability to generate extremely small droplets (2.5 nL volume), deliver outstanding measurement accuracy and achieve rapid reagent transfer rates (approximately 200 Hz) that facilitate swift pipetting operations for various library types [27].

As biological experiments and biomanufacturing technologies are constantly evolving, a variety of innovative solutions provide strong support for the study of single‐cell and complex cell‐loaded structures. Zhang et al. [29] proposed a single‐cell laboratory automation solution that configures a variety of experiments onto a standardized microtube matrix through a precise ultrasonic liquid sample printing technique called PULSE. In a programmable, scalable and biocompatible manner, PULSE enables the transformation of titer plates into droplet arrays (Figure 2D). PULSE enables researchers to perform biological experiments with a single cell as an anchor point, dramatically improving resolution, providing more relevant data for subsequent modelling and downstream analysis, and enabling precise dynamic analysis through automated experiments, making it a powerful tool for biomedical research. In addition, in the field of biomanufacturing, complex cell‐carrying hydrogel structures can be fabricated by changing the bioprinting method, but the existing bioprinter nozzles have many limitations, such as limited printing resolution, easy clogging when the size is less than 100 µm, and the reduction of nozzle diameter will increase the shear stress during printing, triggering mechanical damage and death of cells. To overcome these bottlenecks, Jentsch et al. [30] introduced a novel 3D bioprinting method based on the principle of acoustic droplet printing (ADE). Numerical simulations show that the maximum shear stress in the ADE process is 2.7 times lower than that of the Ø150 µm microvalve nozzle, which can print cell clusters in droplets at millimetre speeds and in single cell‐sized droplets, successfully demonstrating accurate 3D construction of cell‐carrying structures with no negative impact on stem cell morphology, proliferation or differentiation ability (Figure 2E,F). The parameter comparison of each printing method is shown in Table 1.

**TABLE 1:** Performance characteristics of droplet printing modalities.

Droplet printing technology	Printed droplet size common range	Droplet uniformity	System cost	Printing speed	Representative literature
Piezoelectric and thermal drives	pL–µL	Low	Medium	Low	[16, 18]
Pressure drive	fL–nL	Medium	High	Medium	[21, 22]
Electrohydrodynamic drive	aL–fL	High	Low	Medium	[24, 25]
Acoustic drive	pL–µL	High	High	High	[29, 30]

## 3 Droplet Printing Technology Drives Single‐Cell Multimodal Detection

### 3.1 MS Detection

With its high sensitivity, high resolution and multi‐component detection capabilities, MS detection technology has shown unique advantages in the field of single‐cell analysis. This technique enables the simultaneous detection of multiple molecules, such as metabolites, lipids and proteins, in a single cell without labelling, providing a powerful analytical tool for revealing cellular heterogeneity [31]. In particular, the ability to analyse single cells has been significantly improved by coupling microdroplet technology with MS. The microdroplet system can efficiently encapsulate and isolate individual cells to avoid cross‐contamination of samples, while the combination of MS detection enables high‐throughput, high‐precision single‐cell molecular profiling [32]. This combined technology not only improves the throughput of the analysis but also provides more comprehensive molecular information at the single‐cell level, providing important technical support for precision medicine, oncology research and drug development.

#### 3.1.1 Electrospray Ionization MS Detection

**FIGURE 3:**
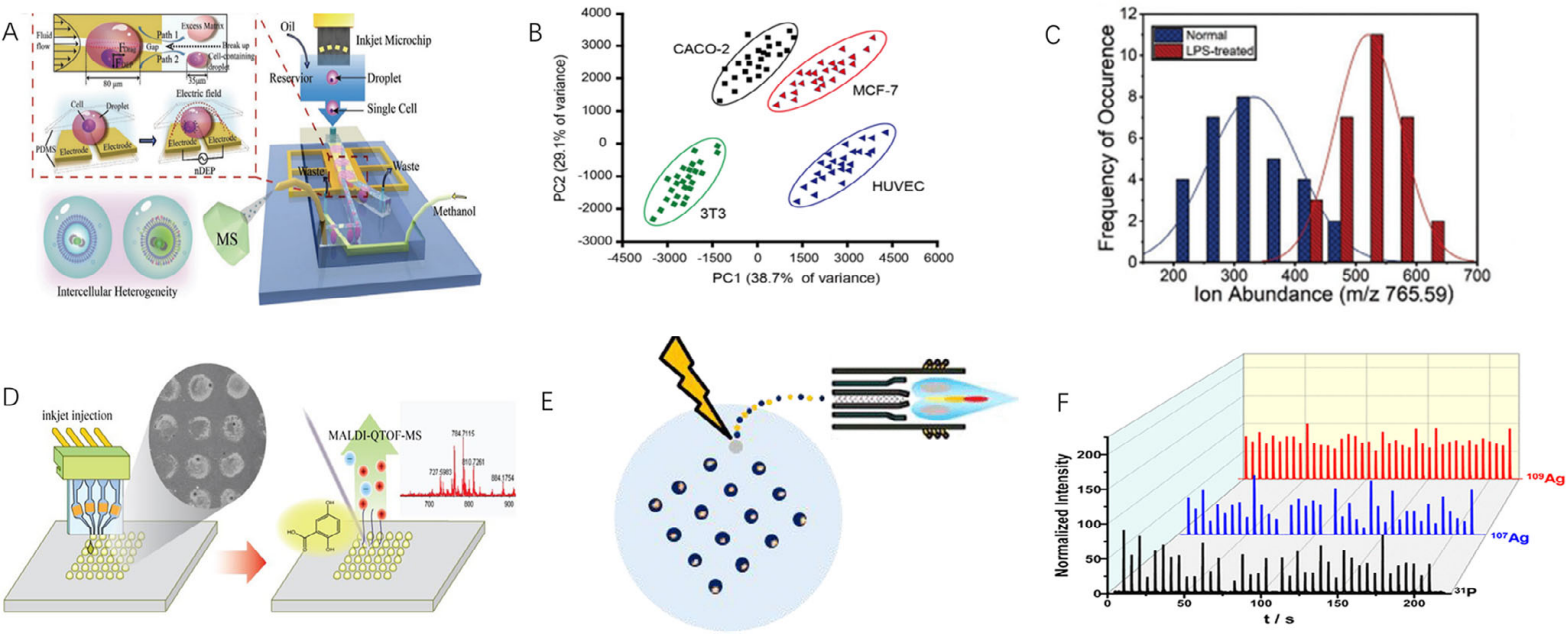
Mass Spectrometry‐Based Single‐Cell Monitoring. (A) Single‐cell assay diagram based on inkjet printing combined with a nano‐ESI device [34]. (B) PCA of different cell types based on PESI analysis [33]. (C) Typing of cells before and after administration [34]. (D) Schematic diagram of inkjet injection and MALDI‐QTOF‐MS [35]. (E) Schematic diagram of SCIDA using LA‐ICP‐MS [37]. (F) P, Ag and Ag transient signals in cells [37].

Electrospray ionization (ESI) technology has unique advantages in single‐cell small molecule detection due to its soft ionization characteristics, high sensitivity and excellent polar small molecule compatibility, making it especially suitable for high‐coverage and accurate quantitative analysis of small molecule compounds such as metabolites and lipids in single cells. Chen et al. [33] developed an inkjet‐printed cell droplet coupled to probe electrospray ionization mass spectrometry (PESI‐MS) technology to achieve efficient single‐cell lipid fingerprinting by accurately depositing cell droplets on tungsten needle tips and spraying them directly (Figure 3B). Combined with a self‐made magnetic stirrer device, the proportion of single‐cell droplets was increased by 43.8%, and the principal component analysis of phospholipid components was used to successfully distinguish eight single‐cell species, providing a simple and reliable method for lipidomics research. In addition, Zhang et al. [34] designed an integrated microfluidic system (Figure 3A). Droplet inkjet printing, dielectric electrophoresis and demulsification technology were integrated to realize online encapsulation, processing and MS detection of single cells. It not only improves the encapsulation rate and reduces matrix interference but also reveals the lipid heterogeneity changes between normal cells and cancer cells, as well as before and after drug treatment, which provides a powerful tool for studying the association between lipid homeostasis and disease (Figure 3C).

#### 3.1.2 Matrix‐Assisted Laser Desorption/Ionization MS Detection

Matrix‐assisted laser desorption/ionization mass spectrometry (MALDI‐MS) has unique advantages in the analysis of macromolecules such as single‐cell proteins and peptides due to its robustness, macromolecule detection ability and excellent spatial resolution, especially suitable for in situ detection and biomacromolecule distribution at the tissue single‐cell level. Korenaga et al. [35] optimized the single‐cell sample introduction method using inkjet technology to achieve precise positioning at the single‐cell and subcellular levels under humidity‐controlled conditions by automatically printing the cell and MALDI matrix solutions onto a hydrophobically modified Indium Tin Oxide glass substrate (Figure 3D). The method provides a new platform for MALDI‐MS analysis by yielding multiple characteristic peaks of phospholipids in cell samples printed in the range of a few hundred microns, achieving the goal of obtaining a wide range of information from single or multiple cells.

#### 3.1.3 Inductively Coupled Plasma MS Detection

Inductively Coupled Plasma Mass Spectrometry (ICP‐MS) has shown irreplaceable advantages in the detection of single‐cell trace elements and metal isotopes due to its ultra‐high sensitivity, wide dynamic range and excellent isotope resolution, especially suitable for single‐cell metallomics research and multi‐element isotope labelling analysis. In addition, the combination of microfluidics and isotope dilution MS further improves the precision of single‐cell quantification [36]. The ‘Single Cell Isotope Dilution Analysis’ (SCIDA) method (Figure 3E) developed by Zheng et al. [37], combined with inkjet printing technology and ICP‐MS, achieved accurate quantification of silver nanoparticle (AgNP) uptake at the single‐cell level, with an average of 396 ± 219 fg Ag per cell measured in 1100 macrophages, and its quantitative results were highly consistent with traditional population analysis. It provides a highly sensitive platform for the study of nanoparticle‐cell interactions (Figure 3F). The downward ICP‐TOF‐MS system developed by Vonderach et al. [38] went beyond the quantitative transport of large microdroplets (up to 90 µm in diameter), with a critical mass of 4.8 pg of carbon for individual beads and cells and an average of 14 and 23 pg of carbon, respectively. The measured diameter of polystyrene beads was in good agreement with the electron microscopy results, and the correlation between the carbon and phosphorus mass in splenocytes was revealed, demonstrating the high accuracy and reliability of the system in the analysis of single cells and microplastics.

In summary, the combination of MS and microfluidics not only expands the dimension of single‐cell analysis but also shows strong application potential in the fields of lipidomics, nanoparticle uptake and microplastics detection.

### 3.2 Surface‐Enhanced Raman Scattering Detection

Surface‐enhanced Raman scattering (SERS) technology can achieve ultra‐high sensitivity and fingerprint detection of biomolecules in single cells through the nanostructure enhancement effect. As a label‐free technique, it is especially suitable for dynamic monitoring of live cells and the analysis of small metabolic molecules. In the field of single‐cell research, SERS technology can break through the sensitivity limitations of traditional optical methods and directly obtain specific vibrational spectra of molecules such as nucleic acids, proteins and metabolites, providing molecular‐level information for the study of cellular heterogeneity [39]. The combination of microdroplets and SERS further enhances single‐cell analysis performance: the microdroplet system can accurately encapsulate single cells and nano‐enhanced substrates to avoid signal cross‐interference; the micro‐scale reaction environment can concentrate the target molecule and significantly enhance the SERS signal intensity. This hyphenated technology combines the efficiency of single‐cell encapsulation, the molecular fingerprinting ability of SERS and the precise control characteristics of microfluidics, providing a new technical path for high‐throughput single‐cell metabolic analysis, drug response monitoring and rare cell screening.

**FIGURE 4:**
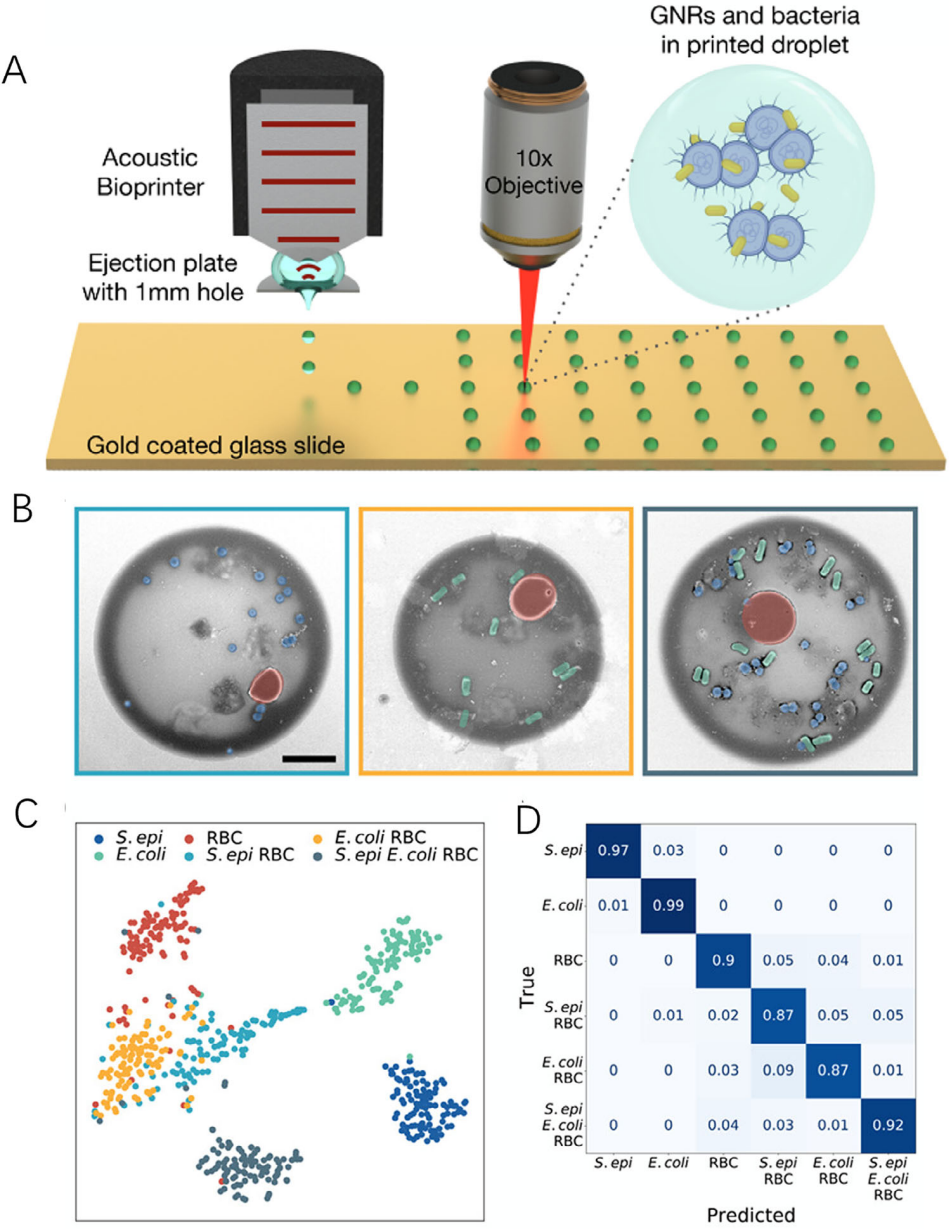
SERS‐Based Single‐Cell Monitoring. (A) Schematic diagram of the acoustic printing platform and confocal Raman [40]. (B) Droplet pseudocolour scanning electron microscope (SEM) image [40]. (C) 2‐component t‐SNE projection of 600 Raman spectra obtained from 100 droplet measurements [40]. (D) Normalized confusion matrix generated from 600 spectra collected from single‐cell line droplets of *Staphylococcus epidermidis*, *Escherichia coli* and mouse erythrocytes mixed with GNR and three cell mixtures using a random forest classifier [40].

Identifying pathogens in complex samples such as blood, urine and wastewater is essential to detect infections and guide optimal treatment. SERS and machine learning (ML) are able to distinguish between multiple pathogen species, but processing complex fluid samples to achieve high sensitivity and specific detection of pathogens remains a major challenge. Here, Safir et al. [40] developed an acoustic bioprinter (Figure 4A) that has the ability to digitize samples into millions of droplets, each containing only a small number of cells, which are identified by SERS and ML. Compared with the limitations of traditional single‐cell analysis methods (e.g., flow cytometry), such as low processing throughput, significant interference from complex sample backgrounds and microfluidic channel clogging risks, this approach effectively overcomes these bottlenecks: digitized droplet processing (1000 droplets/s, 2 pL/droplet) combined with micro‐electro‐mechanical system arrays achieves high throughput (< 1 h for 180 mL samples) and clogging resistance. The experiment demonstrated the rapid printing of 2 pL droplets from *Staphylococcus epidermidis*, *Escherichia coli* and blood (Figure 4B). When these droplets are mixed with gold nanorods (GNRs), up to 1500‐fold SERS enhancement can be obtained. Subsequently, an ML model was trained that achieved more than 99% accuracy for cell‐pure samples and more than 87% for cell‐mixed samples. And more than 90% accuracy was also obtained in droplets with a pathogen‐to‐blood cell ratio of less than 1 (Figure 4C,D). The platform combining bioprinting and SERS accelerates rapid, sensitive detection of pathogens in clinical, environmental and industrial settings.

### 3.3 Ultraviolet and Fluorescence Detection

**FIGURE 5:**
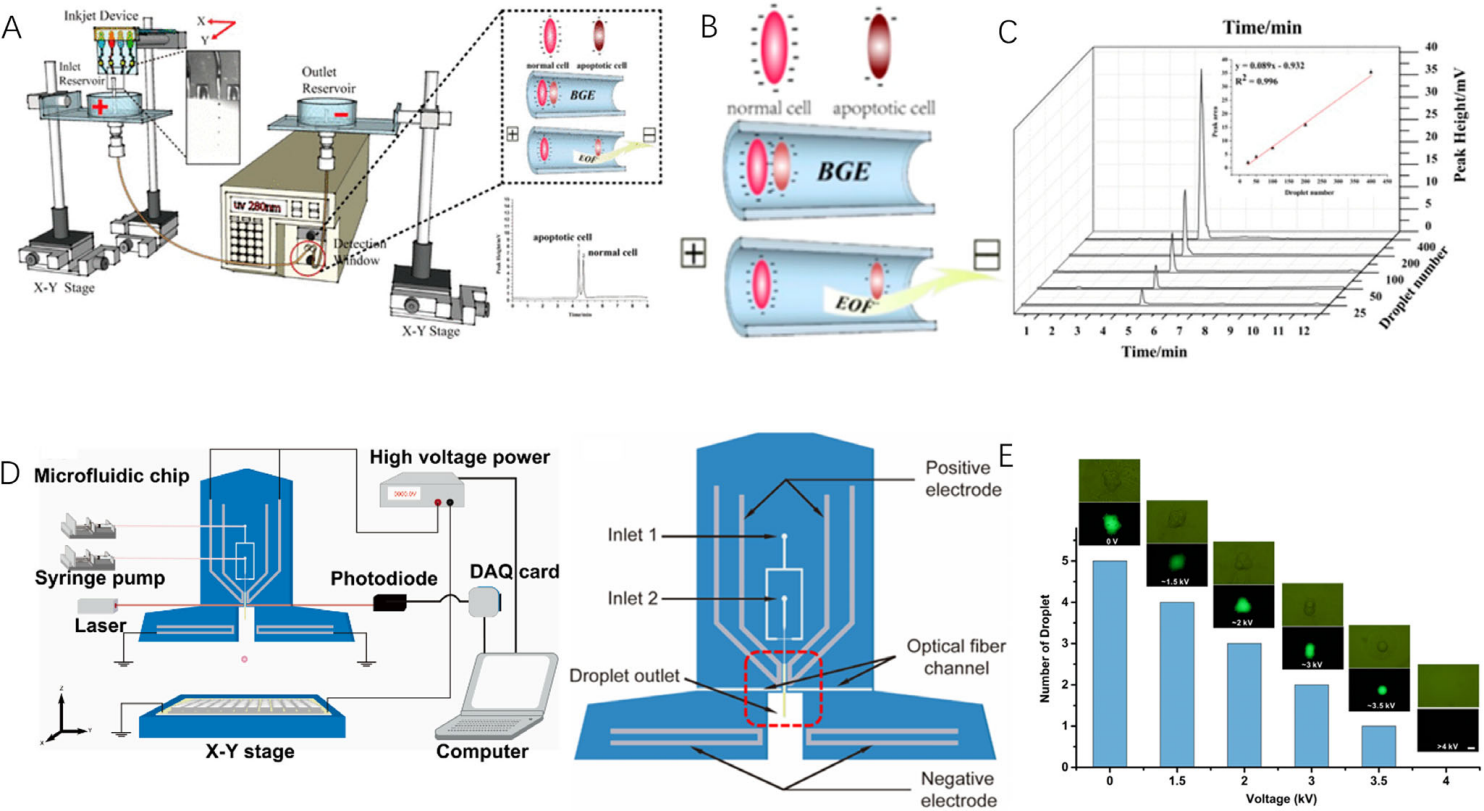
Ultraviolet and Fluorescence‐Based Single‐Cell Monitoring. (A) Diagram of a cell analysis system with capillary electrophoresis [41]. (B) Schematic diagram of cell separation in capillary electrophoresis [41]. (C) Capillary electrophoresis pattern diagram of HepG2 cells with droplets of various numbers 25–400 [41]. (D) Schematic diagram of a microfluidic droplet dispenser system [43]. (E) Scatter plot showing signals from 1 mM sodium fluorescein droplets generated at different voltages in a dispenser [43].

The principle of ultraviolet detection is mainly based on the absorption characteristics of ultraviolet light by the substance. Ultraviolet light is light in the electromagnetic spectrum with wavelengths in the range of 10–400 nm. When ultraviolet light hits a substance, the molecules or atoms in the substance absorb the ultraviolet light of a specific wavelength. This is due to the fact that the electrons in a molecule or atom transition from the ground state to the excited state after absorbing ultraviolet light. Zhang et al. [41] developed an innovative technique (Figure 5A) that combines inkjet printing and capillary electrophoresis to achieve efficient isolation and quantification of mammalian cells. In this method, the on‐demand dispensing of pL droplets is precisely controlled by an inkjet printing system (Figure 5B). After the cell suspension was introduced into the capillary, the separation was achieved based on the difference in cell electrophoretic mobility by zone electrophoresis, and an ultraviolet and visible spectrophotometric detector verified that the peak area had an excellent linear relationship with the number of droplets (25–400 drops) at a cell concentration of 10^6^/mL (*R* ^2^ = 0.996). By optimizing buffer pH (7.4) and electrophoresis conditions, the system successfully isolated normal/apoptotic cells and three different cell lines (HUVEC, HepG2 and Caco‐2). The method could also achieve single‐cell sampling, demonstrating significant advantages in simple operation, accurate quantitation and efficient separation, providing an important tool for cell biology research (Figure 5C).

Fluorescence detection offers unique advantages in single‐cell analysis, where it achieves single‐molecule sensitivity through specific labelling. Microdroplet technology further enhances its performance by using a single droplet as a separate reaction chamber to eliminate cell‐to‐cell interference and concentrate the detection signal, which, combined with high‐speed optical detection, can achieve a throughput of thousands of cells per minute [42]. This hyphenated technology is particularly suitable for single‐cell metabolic analysis, enzyme activity assays and drug response studies, enabling large‐scale single‐cell assays while maintaining high sensitivity. In  an electrohydrodynamic (EHD) microfluidic droplet distribution system (Figure 5D), high‐precision dispensing of individual droplets is achieved by optical signal triggering and EHD drive. The integrated system consists of four modules: chip with integrated pump, optical identification, high‐voltage control and X–Y translation stage, which is optimized to successfully isolate individual *E. coli* cells into petri dishes for subsequent analysis, and its ‘one‐droplet‐one‐well’ dispensing mode realizes automated droplet transfer from the micro‐to‐macro platform (Figure 5E) [43]. This technology platform has demonstrated the unique advantages of precision and automation in the field of monoclonal antibody screening and single‐cell analysis and provides innovative technical solutions for biomedical research.

## 4 Summary and Outlook

The innovative integration of microdroplet printing technology with high‐sensitivity detectors signifies a leapfrogging advancement in single‐cell analysis, shifting the focus from mere ‘tool innovation’ to holistic ‘system integration’. This synergy critically addresses key questions in the field by solving the core challenges of low encapsulation efficiency, strong signal interference and limited throughput. Leveraging a non‐contact active generation mechanism (via air pump, acoustic wave or electrodynamic drive), the microdroplet printing ensures uniform encapsulation while preventing cell damage and enhancing single‐cell capture rates. Its modular design enables in situ coupling with high‐sensitivity detectors [44, 45] (e.g., MS, Raman, fluorescence). This integrated ‘generation‐detection’ approach represents an emerging trend, achieving precise matching between droplet microreactors and detection spots/ion sources. This breakthrough significantly boosts signal acquisition efficiency, eliminates organic phase background noise and enables trace‐level detection sensitivity for single‐cell metabolites and low‐abundance proteins. Consequently, the system not only overcomes the detection bottlenecks of traditional droplet microfluidics but also establishes a critical closed‐loop workflow encompassing single‐cell sorting, in situ lysis and multi‐omics analysis, thereby providing a powerful high‐dimensional analysis tool for studying tumour heterogeneity and the immune microenvironment. Furthermore, artificial intelligence, leveraging its powerful capabilities for high‐dimensional data processing and pattern recognition, has significantly enhanced the efficiency and accuracy of single‐cell data analysis. Combined with the high‐throughput cellular detection data generated by cell printing technology, this AI‐assisted analytical approach provides a more efficient and precise solution for disease diagnosis [40].

Future technology iterations will focus on three major directions: first, the development of an adaptive microdroplet generation system that optimizes droplet size and encapsulation parameters in real time through ML to adapt to the physical characteristics of different types of cells; Second, a multimodal detection‐compatible platform will be constructed, and single‐cell multi‐dimensional omics information will be obtained synchronously by using light‐MS and Raman‐fluorescence confocal technologies. Third, to promote clinical translation and application, through the combination of miniaturized chips and portable detection terminals [46, 47], intraoperative single‐cell drug susceptibility detection or dynamic monitoring of circulating tumour cells can be realized. With the deep integration of micro‐nano manufacturing and ultra‐sensitive sensing technology, the system is expected to make breakthroughs in the frontier fields such as single‐cell dynamic metabolic tracking and spatial multi‐omics integration and ultimately advance the new era of precision medicine from the ‘tissue level’ to ‘single‐cell precision’.

## Conflicts of Interest

The authors declare no conflicts of interest.

## Data Availability

No datasets were generated or analysed during the current study.

## References

[1] W. Chen , O. Guillaume‐Gentil , P. Y. Rainer , et al., “Live‐Seq Enables Temporal Transcriptomic Recording of Single Cells,” Nature 608, no. 7924 (2022): 733–740. 35978187 10.1038/s41586-022-05046-9 PMC9402441

[2] H.‐Y. Wang , Y.‐F. Ruan , L.‐B. Zhu , et al., “An Integrated Electrochemical Nanodevice for Intracellular RNA Collection and Detection in Single Living Cell,” Angewandte Chemie 133, no. 24 (2021): 13352–13358. 10.1002/anie.202014798 33340231

[3] Q. Zhang , C. Zhou , W. Yu , Y. Sun , G. Guo , and X. Wang , “Isotropic Imaging‐Based Contactless Manipulation for Single‐Cell Spatial Heterogeneity Analysis,” TrAC Trends in Analytical Chemistry 157 (2022): 116789.

[4] Y. Shao , Y. Zhou , Y. Wu , et al., “Controllable Fabrication of Pico/Femtoliter Pipette Sampling Probes and Visual Sample Volume Determination,” Talanta 218 (2020): 121096. 32797866 10.1016/j.talanta.2020.121096

[5] M. Labib and S. O. Kelley , “Single‐Cell Analysis Targeting the Proteome,” Nature Reviews Chemistry 4, no. 3 (2020): 143–158. 37128021 10.1038/s41570-020-0162-7

[6] Q. Zhang , Y. Shao , B. Li , et al., “Visually Precise, Low‐Damage, Single‐Cell Spatial Manipulation With Single‐Pixel Resolution,” Chemical Science 12 (2021): 4111–4118. 34163682 10.1039/d0sc05534d PMC8179525

[7] Q. Zhang , Y. Liu , Y. Liang , et al., “Quantitative Analysis of Nanoplastics in Single Cells by Subcellular Chromatography,” Analytical Chemistry 95, no. 26 (2023): 9739–9745. 37347195 10.1021/acs.analchem.2c05428

[8] Y. Shao , Y. Zhou , Y. Liu , et al., “Intact Living‐Cell Electrolaunching Ionization Mass Spectrometry for Single‐Cell Metabolomics,” Chemical Science 13, no. 27 (2022): 8065–8073. 35919431 10.1039/d2sc02569h PMC9278508

[9] M. Yang , F. Wang , H. Liang , et al., “Single‐Cell RNA Sequencing Reveals Distinct Immune Cell Subsets in Phalangeal and Soft‐Tissue Recurrence of Giant Cell Tumor of Bone,” Medicine Advances 1, no. 1 (2023): 14–29.

[10] X. Long , B. Yan , and Z. Mo , “Uncovering the Heterogeneity and Cell Fate Decisions of Endothelial Cells After Myocardial Infarction by Single‐Cell Sequencing,” Medicine Advances 1, no. 3 (2023): 234–245.

[11] W. Chen , B.‐C. Zhang , M. M. Stevanović , L. Huang , and K. Qian , “3D‐Printing Advances Mass Spectrometry in Biomedical Analysis,” LabMed Discovery 1, no. 1 (2024): 100010.

[12] L. Hirt , A. Reiser , R. Spolenak , and T. Zambelli , “Additive Manufacturing of Metal Structures at the Micrometer Scale,” Advanced Materials 29, no. 17 (2017): 1604211. 10.1002/adma.201604211 28052421

[13] D. Foresti , K. T. Kroll , R. Amissah , et al., “Acoustophoretic Printing,” Science Advances 4, no. 8 (2018): eaat1659. 30182058 10.1126/sciadv.aat1659 PMC6118516

[14] Q. Zhang , Z. Toprakcioglu , A. K. Jayaram , G. Guo , X. Wang , and T. P. J. Knowles , “Formation of Protein Nanoparticles in Microdroplet Flow Reactors,” ACS Nano 17, no. 12 (2023): 11335–11344. 37306477 10.1021/acsnano.3c00107 PMC10311583

[15] R. Panneerselvam , H. Sadat , E.‐M. Höhn , A. Das , H. Noothalapati , and D. Belder , “Microfluidics and Surface‐Enhanced Raman Spectroscopy, a Win–Win Combination?,” Lab on a Chip 22, no. 4 (2022): 665–682. 35107464 10.1039/d1lc01097b

[16] K. Zub , S. Hoeppener , and U. S. Schubert , “Inkjet Printing and 3D Printing Strategies for Biosensing, Analytical, and Diagnostic Applications,” Advanced Materials 34, no. 31 (2022): 2105015. 10.1002/adma.202105015 35338719

[17] R. Esfandyarpour , M. J. Didonato , Y. Yang , N. G. Durmus , J. S. Harris , and R. W. Davis , “Multifunctional, Inexpensive, and Reusable Nanoparticle‐Printed Biochip for Cell Manipulation and Diagnosis,” Proceedings of the National Academy of Sciences of the United States of America 114, no. 8 (2017): E1306–E1315. 28167769 10.1073/pnas.1621318114 PMC5338449

[18] A. Ren , F. Chen , C. Ren , et al., “Rapid Screening of Biomarkers in KYSE‐150 Cells Exposed to Polycyclic Aromatic Hydrocarbons via Inkjet Printing Single‐Cell Mass Spectrometry,” Analytical Chemistry 96, no. 31 (2024): 12817–12826. 39052489 10.1021/acs.analchem.4c02332

[19] X. Lin , F. Fang , C. Wang , R. K. Kankala , and S. Zhou , “Inkjet Printing‐Assisted Single‐Cell Microarray on a Hydrophobic Surface Chip for Real‐Time Monitoring of Enzyme Kinetics at Single‐Cell Level,” Talanta 225 (2021): 122019. 33592749 10.1016/j.talanta.2020.122019

[20] L. Chen , Y. Xiao , Q. Wu , et al., “Emulsion Designer Using Microfluidic Three‐Dimensional Droplet Printing in Droplet,” Small 17, no. 39 (2021): 2102579. 10.1002/smll.202102579 34390183

[21] S. Fathi , I. M. Lei , Y. Cao , and Y. Y. S. Huang , “Microcapillary Cell Extrusion Deposition With Picolitre Dispensing Resolution,” Bio‐Design and Manufacturing 6, no. 1 (2023): 1–11. 36644556 10.1007/s42242-022-00205-3 PMC9829649

[22] P. Zhang , L. Xu , H. Chen , and A. R. Abate , “Flow Cytometric Printing of Double Emulsions Into Open Droplet Arrays,” Lab on a Chip 23, no. 10 (2023): 2371–2377. 37070963 10.1039/d3lc00151b PMC12672412

[23] A. Reizabal , B. Tandon , S. Lanceros‐Méndez , and P. D. Dalton , “Electrohydrodynamic 3D Printing of Aqueous Solutions,” Small 19, no. 7 (2023): 2205255. 10.1002/smll.202205255 36482162

[24] W. Zou , H. Yu , P. Zhou , and L. Liu , “Tip‐Assisted Electrohydrodynamic Jet Printing for High‐Resolution Microdroplet Deposition,” Materials & Design 166 (2019): 107609.

[25] L. Wu , S. Xu , J. Wang , et al., “Capillary‐Mediated Single‐Cell Dispenser,” Analytical Chemistry 93, no. 31 (2021): 10750–10755. 34319086 10.1021/acs.analchem.1c01879

[26] Y. Sun , W. Song , X. Sun , and S. Zhang , “Inkjet‐Printing Patterned Chip on Sticky Superhydrophobic Surface for High‐Efficiency Single‐Cell Array Trap and Real‐Time Observation of Cellular Apoptosis,” ACS Applied Materials & Interfaces 10, no. 37 (2018): 31054–31060. 30148358 10.1021/acsami.8b10703

[27] Y. Wang , S. Shaabani , M. Ahmadianmoghaddam , et al., “Acoustic Droplet Ejection Enabled Automated Reaction Scouting,” ACS Central Science 5, no. 3 (2019): 451–457. 30937372 10.1021/acscentsci.8b00782 PMC6439453

[28] C. W. Visser , T. Kamperman , L. P. Karbaat , D. Lohse , and M. Karperien , “In‐Air Microfluidics Enables Rapid Fabrication of Emulsions, Suspensions, and 3D Modular (Bio) Materials,” Science Advances 4, no. 1 (2018): eaao1175. 29399628 10.1126/sciadv.aao1175 PMC5792224

[29] P. Zhang , Z. Tian , K. Jin , et al., “Automating Life Science Labs at the Single‐Cell Level Through Precise Ultrasonic Liquid Sample Ejection: PULSE,” Microsystems & Nanoengineering 10, no. 1 (2024): 172. 39567484 10.1038/s41378-024-00798-y PMC11579414

[30] S. Jentsch , R. Nasehi , C. Kuckelkorn , B. Gundert , S. Aveic , and H. Fischer , “Multiscale 3D Bioprinting by Nozzle‐Free Acoustic Droplet Ejection,” Small Methods 5, no. 6 (2021): 2000971. 10.1002/smtd.202000971 34927902

[31] D. C. Castro , P. Chan‐Andersen , E. V. Romanova , and J. V. Sweedler , “Probe‐Based Mass Spectrometry Approaches for Single‐Cell and Single‐Organelle Measurements,” Mass Spectrometry Reviews 43, no. 4 (2024): 888–912. 37010120 10.1002/mas.21841 PMC10545815

[32] Q. Ruan , J. Yang , F. Zou , et al., “Single‐Cell Digital Microfluidic Mass Spectrometry Platform for Efficient and Multiplex Genotyping of Circulating Tumor Cells,” Analytical Chemistry 94, no. 2 (2021): 1108–1117. 34964350 10.1021/acs.analchem.1c04194

[33] F. Chen , L. Lin , J. Zhang , Z. He , K. Uchiyama , and J.‐M. Lin , “Single‐Cell Analysis Using Drop‐on‐Demand Inkjet Printing and Probe Electrospray Ionization Mass Spectrometry,” Analytical Chemistry 88, no. 8 (2016): 4354–4360. 27015013 10.1021/acs.analchem.5b04749

[34] W. Zhang , N. Li , L. Lin , Q. Huang , K. Uchiyama , and J.‐M. Lin , “Concentrating Single Cells in Picoliter Droplets for Phospholipid Profiling on a Microfluidic System,” Small 16, no. 9 (2020): 1903402. 10.1002/smll.201903402 31769602

[35] A. Korenaga , F. Chen , H. Li , K. Uchiyama , and J.‐M. Lin , “Inkjet Automated Single Cells and Matrices Printing System for Matrix‐Assisted Laser Desorption/Ionization Mass Spectrometry,” Talanta 162 (2017): 474–478. 27837859 10.1016/j.talanta.2016.10.055

[36] X. Zhang , X. Wei , C.‐X. Wu , et al., “Multiplex Profiling of Biomarker and Drug Uptake in Single Cells Using Microfluidic Flow Cytometry and Mass Spectrometry,” ACS Nano 18, no. 8 (2024): 6612–6622. 38359901 10.1021/acsnano.3c12803 PMC10906074

[37] L.‐N. Zheng , L.‐X. Feng , J.‐W. Shi , et al., “Single‐Cell Isotope Dilution Analysis With LA–ICP–MS: A New Approach for Quantification of Nanoparticles in Single Cells,” Analytical Chemistry 92, no. 21 (2020): 14339–14345. 32985178 10.1021/acs.analchem.0c01775

[38] T. Vonderach , A. Gundlach‐Graham , and D. Günther , “Determination of Carbon in Microplastics and Single Cells by Total Consumption Microdroplet ICP‐TOFMS,” Analytical and Bioanalytical Chemistry 416, no. 11 (2024): 2773–2781. 38062197 10.1007/s00216-023-05064-0 PMC11009739

[39] L. Cong , J. Wang , X. Li , et al., “Microfluidic Droplet‐SERS Platform for Single‐Cell Cytokine Analysis via a Cell Surface Bioconjugation Strategy,” Analytical Chemistry 94, no. 29 (2022): 10375–10383. 35815899 10.1021/acs.analchem.2c01249

[40] F. Safir , N. Vu , L. F. Tadesse , et al., “Combining Acoustic Bioprinting With AI‐Assisted Raman Spectroscopy for High‐Throughput Identification of Bacteria in Blood,” Nano Letters 23 (2023): 2065–2073. 36856600 10.1021/acs.nanolett.2c03015 PMC10037319

[41] W. Zhang , N. Li , H. Zeng , H. Nakajima , J.‐M. Lin , and K. Uchiyama , “Inkjet Printing Based Separation of Mammalian Cells by Capillary Electrophoresis,” Analytical Chemistry 89, no. 17 (2017): 8674–8677. 28803473 10.1021/acs.analchem.7b02624

[42] K. J. Bachus , L. Mats , H. W. Choi , G. T. T. Gibson , and R. D. Oleschuk , “Fabrication of Patterned Superhydrophobic/Hydrophilic Substrates by Laser Micromachining for Small Volume Deposition and Droplet‐Based Fluorescence,” ACS Applied Materials & Interfaces 9, no. 8 (2017): 7629–7636. 28169515 10.1021/acsami.6b16363

[43] A. Ge , Z. Diao , Y. Li , et al., “An Integrated Microfluidic Platform for On‐Demand Single Droplet Dispenser With High Accuracy by Electrohydrodynamic (EHD) Printing Technique,” Sensors and Actuators B: Chemical 405 (2024): 135334.

[44] Q. Zhang , B. Chen , Q. Ma , et al., “Single‐Atom Oxide‐Decorated AuNPs for Universal Enhancement in SERS Detection of Pesticide Residues,” Analytica Chimica Acta 1329 (2024): 343192. 39396282 10.1016/j.aca.2024.343192

[45] L. Li , Y. Zhang , L. Zhao , Y. Lv , F. Qu , and Q. Ma , “In‐Capillary Aptamer‐Functionalized Dispersive Solid‐Phase Microextraction for Dynamic Transfer Enrichment and Miniature Mass Spectrometry Analysis: A Magnetically Driven Capture‐and‐Release Strategy,” Chemical Engineering Journal 485 (2024): 149997.

[46] Z. Ju , X. Guo , L. Li , et al., “Improved Point‐of‐Care Mass Spectrometry Analysis With Thin‐Layer Chromatography‐Based Two‐Dimensional Separation and Spray Ionization,” Analytical Chemistry 97, no. 1 (2025): 712–720. 39722213 10.1021/acs.analchem.4c05129

[47] X. Zhu , Q. Zhang , X. Qi , et al., “Unraveling the Structural Evolution of Silver Plasmonic Hotspots for the Detection of Oxidative ONOO—Radicals via SERS Probe Decay,” Microchimica Acta 192 (2025): 182. 39992489 10.1007/s00604-025-07045-9

